# Supplementary material for: Inhibition of inflammatory osteoclasts accelerates callus remodeling in osteoporotic fractures by enhancing CGRP+TrkA+ signaling
Source: Cell Death Differ. 2024 Sep 2;31(12):1695–706. doi: 10.1038/s41418-024-01368-5 (PMC11618598; doi:10.1038/s41418-024-01368-5)
Supplement: Supplementary file 1 — Supplemental information [file 41418_2024_1368_MOESM1_ESM.pdf]

Supplemental Information Legends

Figure.S1. Reduced bone mass and delayed fracture healing in OVX mice.

Figure.S2. Conditional knockout of $CX3CR1^{+}$iOCs accelerates callus remodeling by enhancing CGRP+TrkA+ signaling.

Figure.S3. Sema3A secreted by $Cx3cr1^{+}$iOCs inhibits $CGRP^{+}TrkA^{+}$ sensory nerve signaling via GSK3β-Akt pathway in vitro.

Fig.S4. Targeting Sema3A in $Cx3cr1^{+}$iOCs enhances $CGRP^{+}TrkA^{+}$ signaling and promotes callus remodeling.

Fig.S5. Elevated $Cx3cr1^{+}$iOCs and diminished $CGRP^{+}TrkA^{+}$ Signaling in osteoporotic fracture tissue samples.

Figure.S6. Western blot original bands

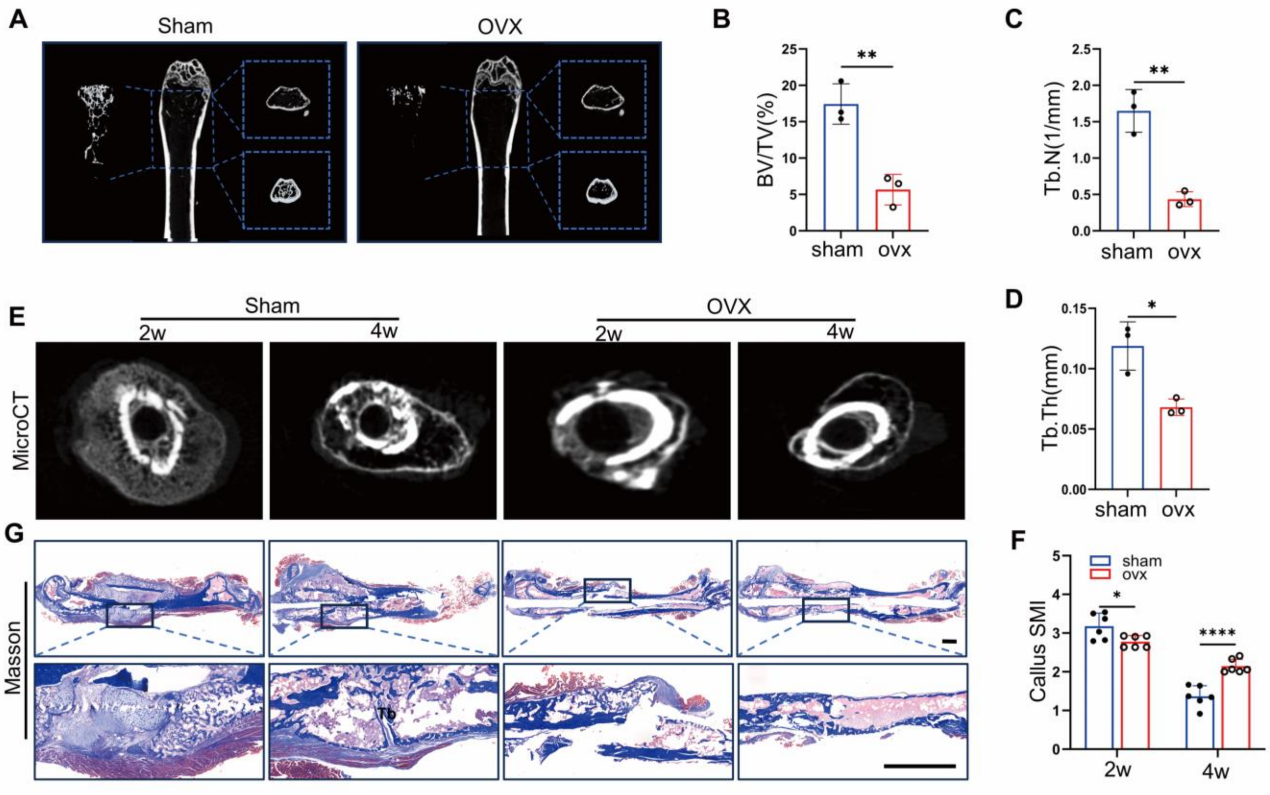

Figure.S1. Reduced bone mass and delayed fracture healing in OVX mice.

(A) Representative Micro-CT images of femurs in sham and OVX mice. Scale bar: 1 mm.

(B-D) Micro-CT quantitative analysis of the femurs from (A). The Micro-CT parameters include bone volume fraction (BV/TV) of calluses, Tb.Th (trabecular thickness), Tb.N (trabecular number). n=3.

(E) Representative micro-CT images of femoral fractures at 2- and 4-weeks post-fracture.

(F) Micro-CT quantitative analysis of the callus from (E). The micro-CT parameters include the structure model index (SMI). n=6.

(G) Representative images of Masson staining of femoral fractures in sham and OVX mice at 2- and 4-weeks post-fracture. Upper panels display global views; lower panels show close-up views of the fracture sites. Tb: trabecular bone. Scale bar: 1 mm. Data presented as mean ± SEM. *P < 0.05, **P < 0.01, ***P < 0.001, ****P < 0.0001.

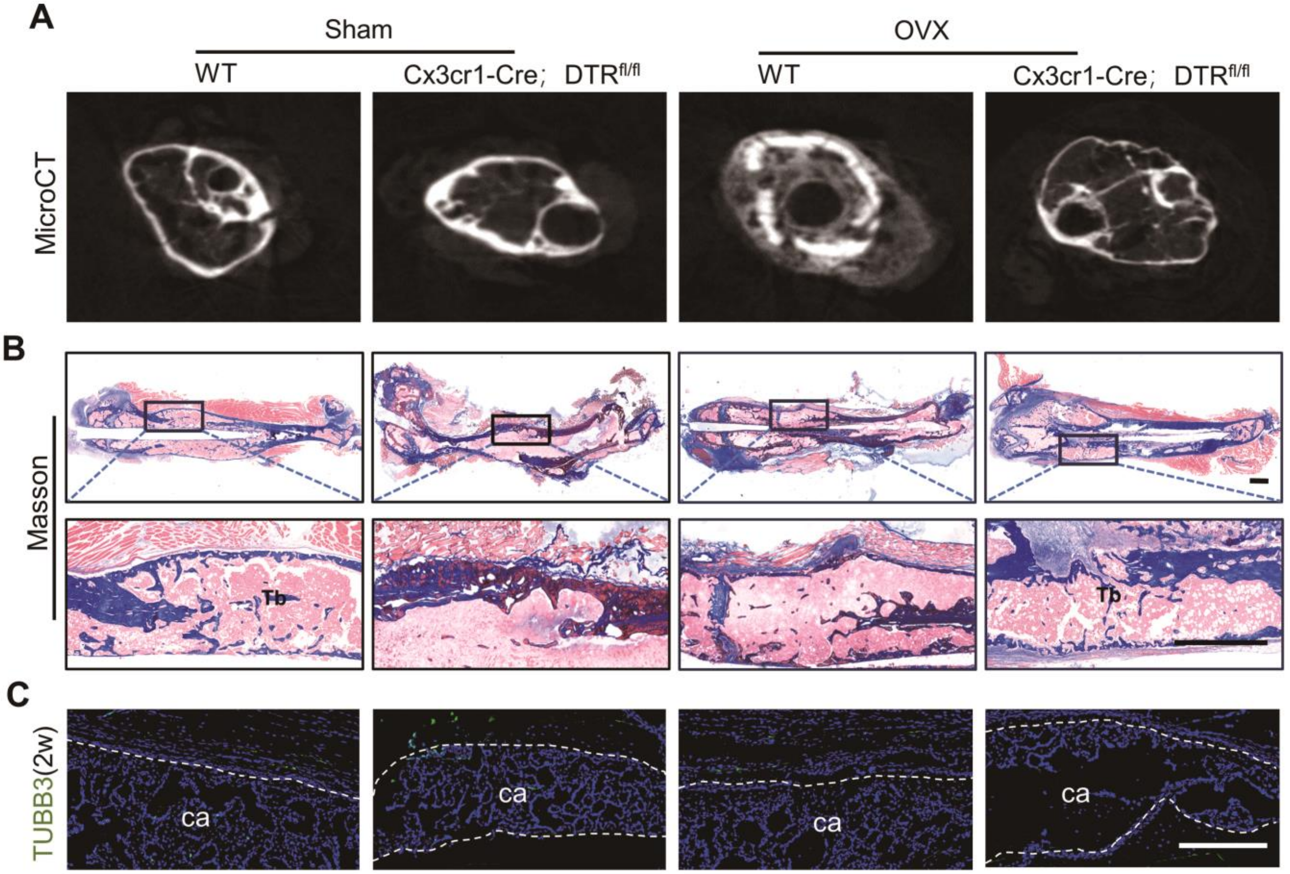

Figure.S2. Conditional knockout of CX3CR1⁺iOCs accelerates callus remodeling by enhancing CGRP+TrkA+ signaling.

(A) Representative micro-CT images of femoral fractures of Cx3cr1-Cre; $DTR^{fl/fl}$ mice and WT littermates at 4 weeks post-fracture.

(B)Representative images of Masson staining of the femoral fractures of Cx3cr1-Cre; $DTR^{fl/fl}$ mice and WT at 4 weeks post-fracture. The upper panels display global views, while the lower panels show close-up views of the fracture sites. Tb: trabecular bone. Scale bar: 1 mm.

(C) Immunofluorescence staining images showing TUBB3 protein levels in calluses from Cx3cr1-Cre; $DTR^{fl/fl}$ mice mice and WT littermates at 2 weeks post-fracture. ca: callus. Scale bar: 200 μm. Data presented as mean ± SEM. *P < 0.05, **P < 0.01, ***P < 0.001, ****P < 0.0001.

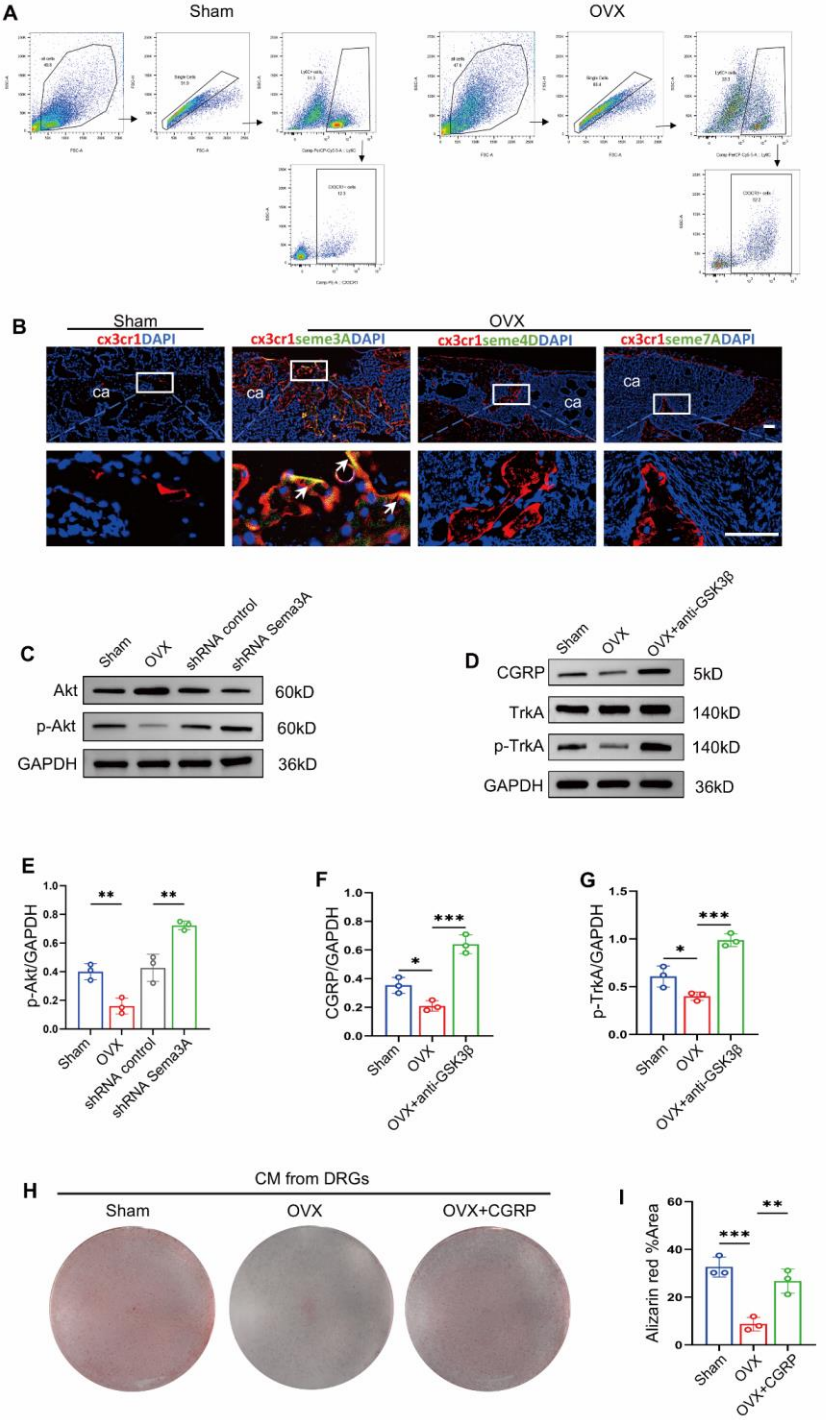
A
Sham
OVX
B
Sham
OVX
cx3cr1DAPI
cx3cr1seme3ADAPI
cx3cr1seme4DDAPI
cx3cr1seme7ADAPI
ca
C
Sham
OVX
shRNA control
shRNA Sema3A
Akt
p-Akt
GAPDH
60kD
60kD
36kD
D
Sham
OVX
OVX+anti-GSK3β
CGRP
TrkA
p-TrkA
GAPDH
5kD
140kD
140kD
36kD
E
p-Akt/GAPDH
F
CGRP/GAPDH
G
p-TrkA/GAPDH
H
CM from DRGs
Sham
OVX
OVX+CGRP
I
Alizarin red %Area

Figure.S3. Sema3A secreted by Cx3cr1$^+$iOCs inhibits CGRP$^+$TrkA$^+$ sensory nerve signaling via GSK3β-Akt pathway in vitro.

(A) Flow cytometry analysis of Cx3cr1$^+$Ly6C$^+$ cells in sham and OVX mouse bone marrow. n=3.

(B) Representative coimmunostaining images of CX3CR1, Sema3A, Sema4D, and Sema7A, and quantification in calluses from sham and OVX mice at 4 weeks post-fracture. ca: callus. white arrows point to Cx3cr1$^+$ Sema3A$^+$. Scale bar: 200 μm.

(C and E) Western blot analysis of Akt and p-Akt in DRGs treated with or without Sema3A from by cx3cr1$^+$iOCs.

(D) Western blot analysis of CGRP, TrkA, p-TrkA in DRGs treated with Sham, OVX or OVX+anti-GSK3β.

(F-G) Quantitative analysis of CGRP, TrkA and p-TrkA expression from (D).

(H-I) Alizarin Red staining and quantitative analysis of PCs differentiation into osteoblasts， with or without CGRP signaling. n = 3 per group; data presented as mean ± SEM. *P < 0.05, **P < 0.01, ***P < 0.001 and ****P < 0.0001.

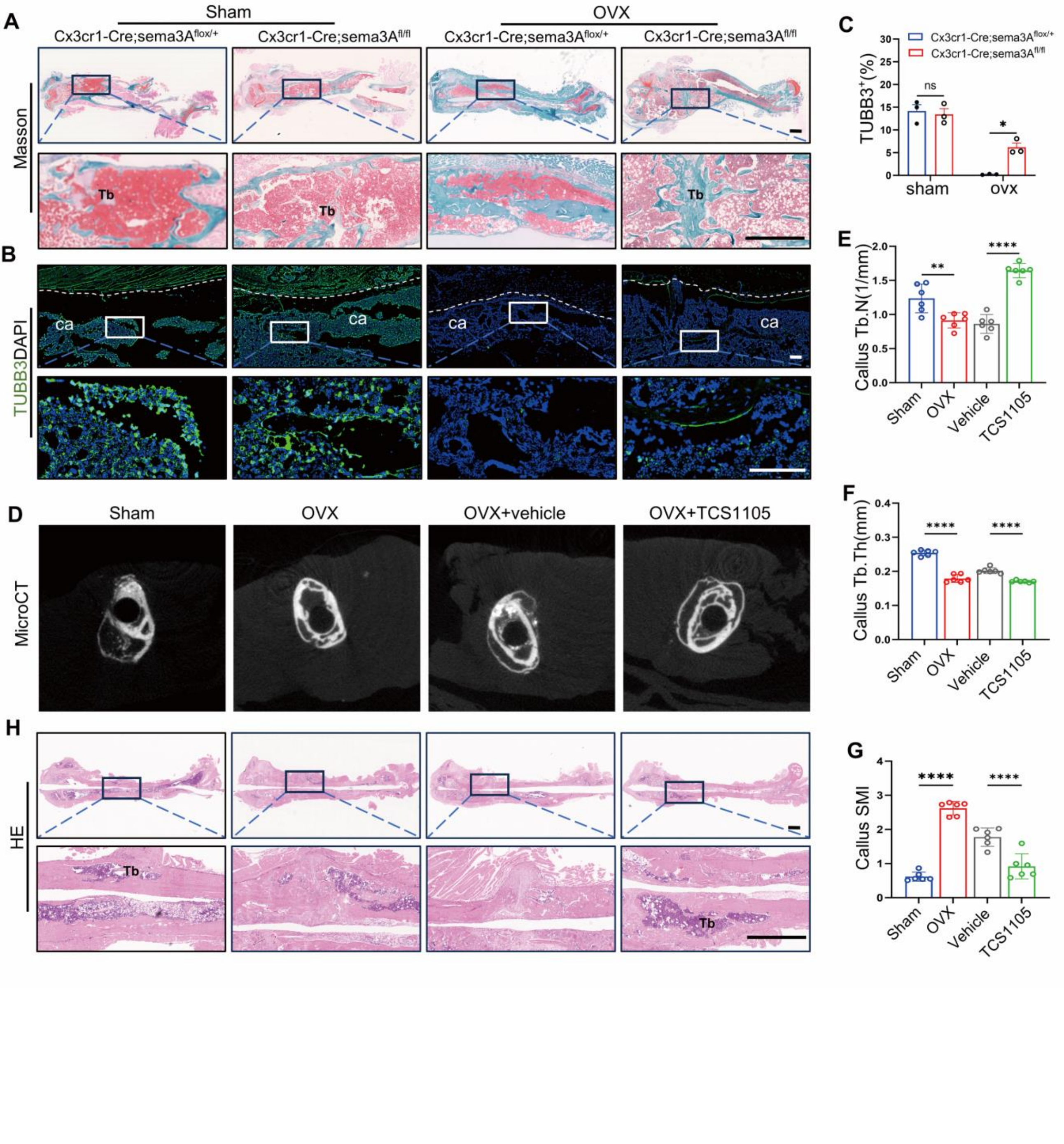

A
Sham
OVX
Cx3cr1-Cre;sema3A$^{flox/+}$
Cx3cr1-Cre;sema3A$^{fl/fl}$
Masson
Tb
B
TUBB3DAPI
ca
C
Cx3cr1-Cre;sema3A$^{flox/+}$
Cx3cr1-Cre;sema3A$^{fl/fl}$
TUBB3^{+}(%)
ns
*
sham
ovx
D
Sham
OVX
OVX+vehicle
OVX+TCS1105
MicroCT
E
Callus Tb.N(1/mm)
**
****
Sham
OVX
Vehicle
TCS1105
F
Callus Tb.Th(mm)
G
Callus SMI
H
HE

Fig.S4. Targeting Sema3A in Cx3cr1$^+$iOCs enhances CGRP$^+$TrkA$^+$ signaling and promotes callus remodeling.

(A) Representative SO-FG-stained images of femoral fractures in Cx3cr1-Cre; Sema3A$^{fl/fl}$ OVX mice and their littermate controls at 4 weeks post-fracture. Upper panels display global views; lower panels show close-up views of the fracture sites. Tb: trabecular bone. Scale bar: 1 mm.

(B-C) Immunofluorescence staining images and quantification of TUBB3 protein levels in calluses from Cx3cr1-Cre; Sema3A$^{fl/fl}$ OVX mice and their littermate controls at 4 weeks post-fracture. ca: callus. White dashed lines outline the callus boundaries. Scale bar: 200 μm. n=3.

(D) Representative micro-CT images of femoral fractures of WT mice treated with TCS1105 or vehicle at 4 weeks post-fracture. Scale bar: 1 mm.

(E-G) Micro-CT quantitative analysis of the callus from (D). Parameters include trabecular thickness (Tb.Th), trabecular number (Tb.N), and structure model index (SMI). n=6.

（H）Representative images of H&E staining of femoral fractures in WT mice treated with Sema3A inhibitor or normal saline at 4 weeks post-fracture. Upper panels display global views; lower panels show close-up views of the fracture sites. Tb: trabecular bone. Scale bar: 1 mm. Data presented as mean ± SEM. *P < 0.05, **P < 0.01, ***P < 0.001, ****P < 0.0001.

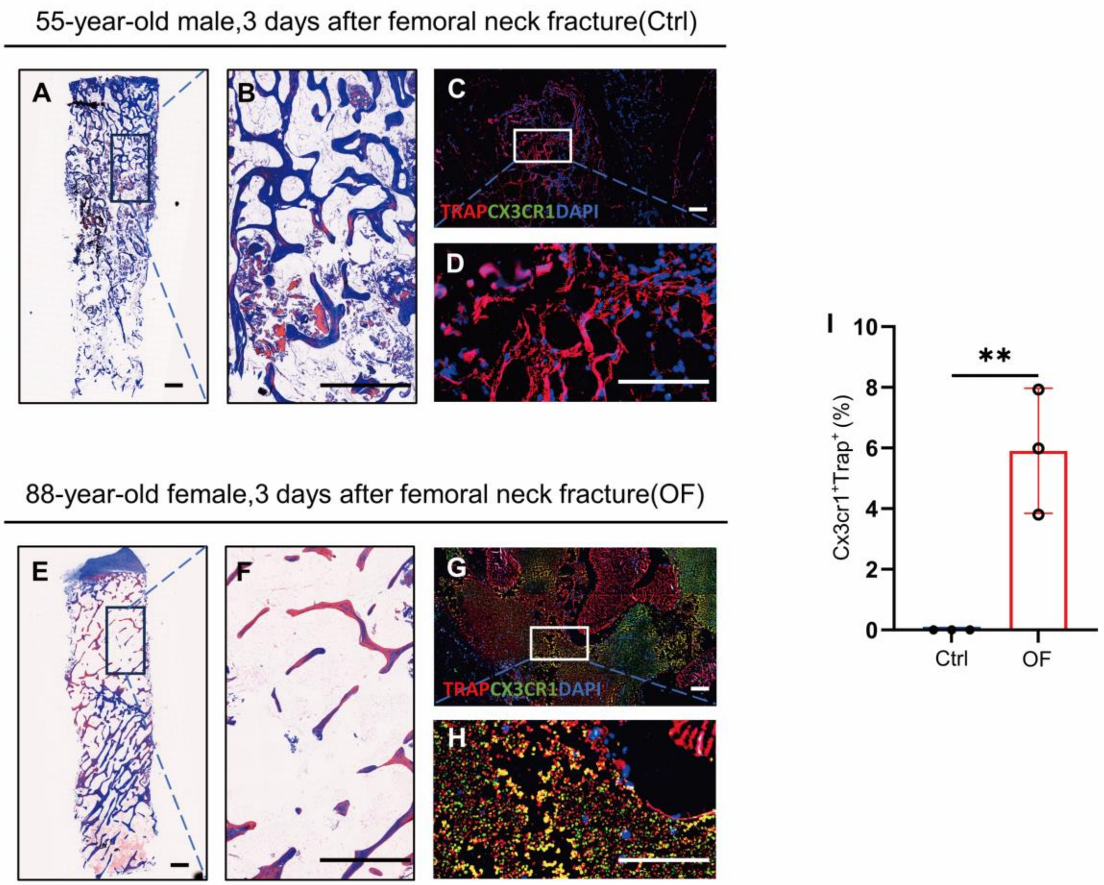

Fig.S5. Elevated Cx3cr1^{+}iOCs and diminished CGRP^{+}TrkA^{+} Signaling in osteoporotic fracture tissue samples.

(A-D) Pathological analysis of a femoral neck fracture in a 55-year-old male. Representative images of Masson staining (A) and detailed images (B) on the left. Immunofluorescence staining of CX3CR1 and TRAP coimmunostaining (C), and detailed images (D) on the right. Scale bar: 1 mm.

(E-H) Pathological analysis of a femoral neck fracture in an 88-year-old female. Representative images of Masson staining (E) and detailed images (F) on the left. Immunofluorescence staining of CX3CR1 and TRAP coimmunostaining (G), and detailed images (H) on the right. Scale bar: 1 mm.

(I) Quantitative analysis of CX3CR1 and TRAP fluorescence intensity in fracture tissue samples from (C) and (G). n = 3 per group; data presented as mean ± SEM. *$P < 0.05$, **$P < 0.01$, ***$P < 0.001$ and ****$P < 0.0001$.

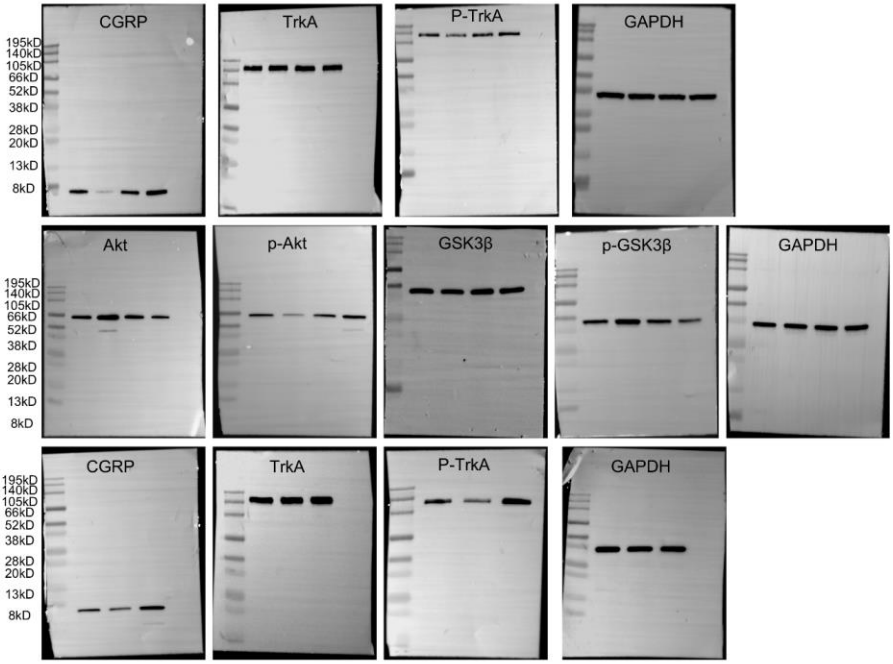

Figure.S6. Western blot original bands
